# Mapping the Origins of Time: Scalar Errors in Infant Time Estimation

**DOI:** 10.1037/a0037108

**Published:** 2014-06-30

**Authors:** Caspar Addyman, Sinead Rocha, Denis Mareschal

**Affiliations:** 1 Centre for Brain and Cognitive Development, Birkbeck, University of London

**Keywords:** interval timing, time perception, infancy

## Abstract

Time is central to any understanding of the world. In adults, estimation errors grow linearly with the length of the interval, much faster than would be expected of a clock-like mechanism. Here we present the first direct demonstration that this is also true in human infants. Using an eye-tracking paradigm, we examined 4-, 6-, 10-, and 14-month-olds’ responses to the omission of a recurring target, on either a 3- or 5-s cycle. At all ages (a) both fixation and pupil dilation measures were time locked to the periodicity of the test interval, and (b) estimation errors grew linearly with the length of the interval, suggesting that trademark interval timing is in place from 4 months.

Interval timing concerns our ability to judge, compare, and reproduce time intervals for durations from about half a second to a few minutes (Grondin, 2008; Zakay & Block, 1997). It is fundamental to the way we structure our social and physical interactions with the world. One surprising thing about our interval timing abilities (which contrast sharply with precision timing involved in motor control) is how bad they are. Errors in our time estimates grow linearly with the length of the interval, much faster than would be expected of a repetitive clock-like mechanism (Hass & Hermann, 2012). This so-called scalar property of interval timing is shared by humans, rats, and pigeons (Gibbon, 1977) and can even be found in rate dependent habituation in *C. elegans* (Staddon & Higa, 1996). Interval timing is typically investigated in human adults and rats by requiring them to reproduce a target interval. However, it has never been directly demonstrated in infants because of the difficulty of getting them to generate repeated estimates of fixed intervals.

Timing abilities develop slowly from birth. Clifton (1974) established that even neonates could show a response (heart rate deceleration) to an omitted timed event. This pattern was also found at 2 months old (Boswell, Garner, & Berg, 1994) and 4 months old (Colombo & Richman, 2002). Heart rate measures sometimes also show anticipation developing from 4 months onward, but the data quickly become difficult to interpret (Boswell et al., 1994; Donohue & Berg, 1991). Studies using visual habituation paradigms have shown that infants’ ability to discriminate temporal intervals increases from a ratio 1:2 at 6 months (vanMarle & Wynn, 2006) to a ratio of 2:3 at 10 months (Brannon, Suanda, & Libertus, 2007), mirroring the development of numerical discrimination (Lipton & Spelke, 2003). Measurement of event-related potentials (ERPs) reveals that 10-month-olds show brain responses to omitted stimuli similar to those of adults (Brannon, Libertus, Meck, & Woldorff, 2008; Brannon, Roussel, Meck, & Woldorff, 2004). Although a sensitivity to regular timings seems to be present from early infancy, as evidenced above, timing accuracy continues to develop well into childhood (e.g., Droit-Volet, Tourret, & Wearden, 2004; Friedman, 2008; Goldberg, 1995).

The existing research on infant timing has some methodological limitations. Many previous studies of infant time estimation have relied on violation of expectation paradigms with binary measures of novelty and familiarity or habituation and dishabituation, which provide limited information (Aslin, 2007). In particular, they do not allow the detection of time-locked behaviors. Heart rate variability (e.g., Donohue & Berg, 1991) does provide a temporal signal, but this is of low resolution (0.5 Hz) and is subject to rapid habitation, which makes data difficult to interpret. ERPs (e.g., Brannon et al., 2008) have high temporal resolution but require extensive repetition with averaging over large numbers of trials, and can produce noisy data that are difficult to analyze and interpret. There is also a theoretical difficulty in that ERPs need a clear external “event” marker to line up multiple trials. This makes it less suitable for studying subjective interval timing where the events of interest are internally generated and have substantial temporal variability. Finally, none of these studies directly address infants’ ability to anticipate and act on temporally predictable events.

In the present study we use an eye-tracking system to provide online measures of infant voluntary and involuntary responses in an interval timing task. We use a “peek-a-boo” animation in which a cartoon character pops up on the screen at regular intervals (every 3 s in one condition, 5 s in another) accompanied by a socially engaging sound effect (adult female voice addressing the infant). The infants saw seven repetitions of this event followed by a blank screen for a further 6 or 10 s, respectively. Modern eye trackers provide an accurate measure of fixation location and pupil diameter with high temporal resolution. Fixation data can show where infants look, when they saccade, and for how long they fixate. This provides a measure of voluntary response to expectations about the timing of events. In contrast, changes in pupil diameter are involuntary and accompany violations of expectation in infants (Jackson & Sirois, 2009). They can also provide a proxy for attention or cognitive load (Laeng, Sirois, & Gredebäck, 2012). Responses in both modalities provide estimates of the infant’s repeated subjective judgments of the duration of the interval between predictable stimuli.

## Method

### Participants

Nineteen four-month-olds (11 female; mean age = 133 days; range: 118–144 days), 20 six-month-olds (5 female; mean age = 181 days; range: 166–197 days), 20 ten-month-olds (14 female; mean age = 308 days; range: 293–320 days), and 20 fourteen-month-olds (10 female; mean age = 433 days; range: 415–447 days) took part in the 5-s condition.

Seventeen four-month-olds (9 female; mean age = 133 days; range: 118–144 days), 12 six-month-olds (5 female; mean age = 186 days; range: 166–215 days), 13 ten-month-olds (6 female; mean age = 308 days; range: 291–323 days), and 13 fourteen-month-olds (6 female; mean age = 428 days; range: 419–447 days) took part in the 3-s condition.

Of these, 35 infants (13 four-month-olds, 9 six-month-olds, 10 ten-month-olds, and 3 fourteen-month-olds) took part in both conditions. These infants took part in the 5-s condition first, had a 10-min break, then completed the 3-s condition. Finally, across both conditions 29 infants were excluded: seven infants because of fussiness, four because of experimenter error or computer failure, and 18 due to insufficient eye-tracking data.

### Apparatus

Stimuli were presented on a Tobii T120 eye-tracking monitor with a 17-in. (43.18-cm) screen, presentation was controlled by an Apple PowerMac OS X Intel 2.8 Ghz, running MATLAB R2009b (MathWorks Ltd.) with Psychtoolbox (Brainard, 1997; Pelli, 1997).

### Stimuli and Design

**Figure 1 fig1:**
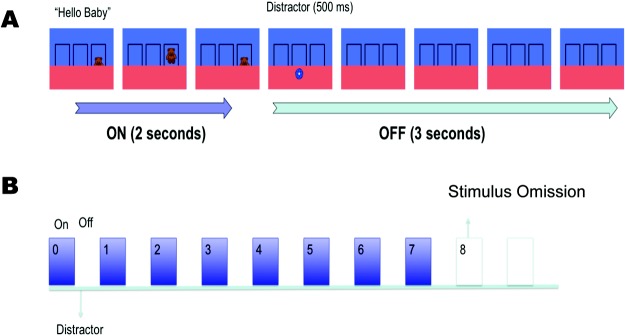
Schematic representation of stimulus presentation in the 5-s condition. (A) In one on–off cycle the target rose into the target area accompanied by a vocal prompt. It remained stationary for 1 s before disappearing again. A rapidly shrinking distractor then briefly appeared, and the frame remained empty for the remainder of the period. (B) Each block consisted of seven cycles with the character reappearing at fixed intervals followed by a longer test period in which nothing happened. See the online article for the color version of this figure.

Each condition (5- or 3-s intervals) consisted of three blocks of eight learning trials in which a cartoon character moved up from behind an occluder into a target area directly above the occluder and then back down again, with a periodicity of either 5 s or 3 s (see Figure 1). This was followed by a “missed beat” in which the animated character did not appear and the screen remained unchanged. The missed beat lasted twice as long as a regular beat, thereby enabling us to assess responses occurring after the expected appearance of a character.[Fig-anchor fig1]

Each block featured one of six different cartoon characters (either one of two teddy bears, one of two dinosaurs, a fish, or a tiger), and each character was paired with a single verbal prompt (“Coo-ee!,” “Hey, Baby,” then “What’s that?”). The three target areas (defined by black borders) were located to the left, to the right, or at the center of the screen. The target area for the character remained the same for all the three blocks. For those infants participating in both conditions, both the target area and set of characters were changed between conditions.

In the 5-s trials, a complete beat cycle consisted of the character moving from behind the occluder to the target area in 500 ms accompanied by a verbal prompt. It then remained stationary for 1,000 ms before moving back behind the occluder in 500 ms. The screen then remained unchanged for 3 further seconds. This blank interval was cut to 1 s in the 3-s cycle condition. In both conditions a distractor (an orange star or a blue circle rapidly decreasing in size) was shown on the lower part of the screen after the character had disappeared. This was to stop infants remaining fixated on the target area.

All animated characters were of comparable size (6° of visual angle). The luminosity of the screen did not significantly differ when the stimulus was present or absent, nor did it differ between characters (see Figure S1 in supplemental materials).

### Procedure

Each infant was seated on his or her carer’s lap, approximately 60 cm from the eye tracker screen. A 5-point calibration sequence was run until at least 4 points were properly calibrated for each eye. In addition, 61 of the infants took part in a simple habituation-to-a-checker-board task before and after testing as part of a separate study.

## Results

For each infant the eye tracker recorded fixation and pupil diameter information at a sampling rate of 60 Hz. Because eye-tracking data, especially with infants, can be intermittent, we analyzed the data by fitting smoothed curves to both the fixation and pupil data as described below (see also Jackson & Sirois, 2009; Ramsay & Silverman, 2002).

To normalize the raw data from the eye tracker and allow for comparison between participants, we first divided the data into fixed 50-ms bins. Fixation distance was calculated as the linear distance (in pixels) from the center of the target area to the mean fixation point found by averaging fixation data for left and right eyes (if available), or for a single eye, if only one eye was available. Pupil diameter values were similarly combined. We excluded blocks of trials in which there was tracking data for less than 50% of the total presentation time. A total of 92/284 (.32) where excluded in the 5-s condition and 39/171 (.23) in the 3-s condition. The remaining data still had an average of .26 missing samples. Missing values were linearly interpolated, and a low-pass filter was applied with the “filter” function built into MATLAB (R2009b) with a tau parameter of 0.15.

Next, these data were fit with a piecewise smooth B-spline function of order 4 with 1 base per second to give either 30 bases (3-s condition) or 50 bases (5-s condition). This value smoothed the data while preserving the major event related peaks (Ramsay & Silverman, 2002). It gives our B-spline functions a comparable temporal resolution to Jackson and Sirois (2009), who fitted a 26-s presentation with 24 bases (1.08 bases per second). The resulting continuous time series data are shown in Figure S1 in the supplemental materials.

**Figure 2 fig2:**
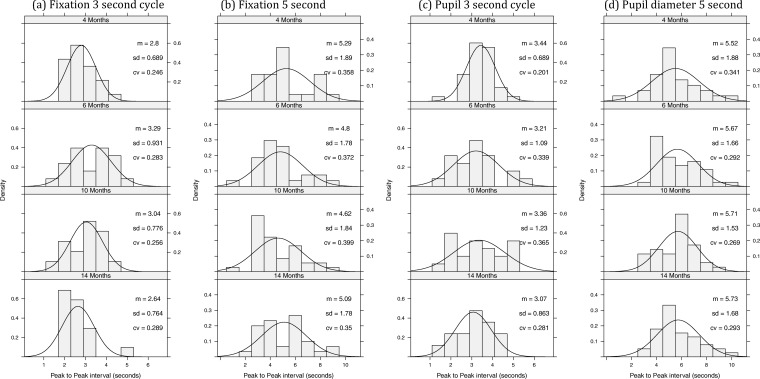
Histograms of the peak-to-peak value for fixation distance and pupil diameter as a function of timing condition and age. In general, median distribution values are close to 5 in the 5-s conditions and 3 in the 3-s conditions. cv = coefficient of variation.

To investigate infants’ responses to the missed beat, we analyzed the peak-to-peak measurements (for both fixation distance and pupil dilation) for the value associated with the last occurrence of a visible target and the value associated with the missed beat in which the screen remains blank (see Figure S2 in the supplemental materials). For fixation distance this corresponds to the distance between minima, whereas for pupil size this corresponds to distance between maxima. The second peak was assumed to be associated with infants’ expectation of the target reappearing at a predictable time, and the interval between the peaks was taken as a measure of the infants’ subjective time estimate of when the target should reappear. In other words, it is a direct measure of the infants’ temporal interval judgments. Figure 2 plots histograms for peak-to-peak fixation and pupil diameter grouped by age and time interval.[Fig-anchor fig2]

Approximately 55% of fixation data and 66% of pupil data yielded interval estimates via this method. For each data type we ran an analysis of variance with age and time interval as between-subject factors.^1^ For fixation, we found that estimates clearly varied according to the time interval, *F*(1, 192) = 82.4, *p* < .0001, η^2^ = .30, with no effect of age, *F*(3, 192) < 1, and no interaction, *F*(3, 192) = 1.48, *p* < .22. For pupil diameter, we found that estimates clearly varied according to the time interval, *F*(1, 233) = 140.5, *p* < .0001, η^2^ = .38, with a trend toward a main effect of age, *F*(3, 233) = 2.3, *p* < .08, and no interaction, *F*(3, 233) < 1. The lack of an age effect shows that interval timing ability is present as young as 4 months of age. In both cases, data failed Levene’s test for homogeneity of variance: fixation, *F*(7, 192) = 5.62, *p* < .0001; pupil data, *F*(7, 232) = 4.19, *p* < .0002. This suggests, in line with the scalar property of interval timing, that variance increases with length of the target interval. However, this breaks the homogeneity of variance assumptions of analysis of variance. Consequently, even though *t* and *F* tests with near equal *n*s are generally robust to variations in variance across samples (Boneau, 1960), we also looked at relative time data. All infant time estimates for the 3- and 5-s conditions were divided by 3 and 5, respectively, and the above analyses were rerun. Levene’s tests were no longer significant: for fixation, *F*(7, 192) = 2.18, *p* < .09; and for pupil, *F*(7, 232) = 1.52, *p* < .16. For fixation, time no longer had a significant difference between groups, *F*(1, 192) = .01, *p* < .94, η^2^ < .0001, with no effect of age, *F*(3, 192) < 1, and no interaction, *F*(3, 192) = 2.18, *p* < .09. For pupil diameter, time was no longer significant, *F*(1, 232) = 0.24, *p* < .62, η^2^ = .001, and there were no other effects (*F*s < 1). Taken together, these analyses support the idea that infant time estimates approximate the target interval with linear growth in error.

**Figure 3 fig3:**
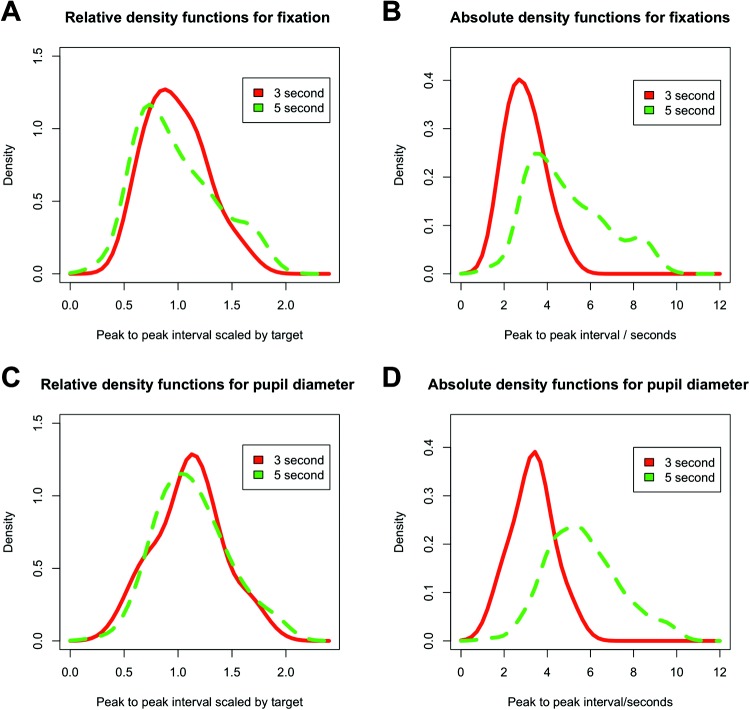
Density functions for infants’ time interval estimates. Figures 3A and 3C show relative peak-to-peak scores for all infants scaled by the target interval for fixation and pupil diameter. Figures 3B and 3D represent the same data plotted on an absolute scale. See the online article for the color version of this figure.

Finally, we combined data across all ages in order to plot density distributions of estimates (see Figure 3). Peak-to-peak distances for both the fixation distance and the pupil dilation were modulated by the time interval. In both cases, the value closely approximated the interval (3 s or 5 s) of the test condition, showing that infants’ interval estimates were tuned to the particular intervals that they experienced. In addition, their errors (the deviation of the their estimates from the actual interval values) grew in proportion to the length of the target interval (Figures 3B and 3D). When the individual peak-to-peak estimates are divided by the actual target interval length (3 or 5, respectively), the curves have the same shape (Figures 3A and 3C). The curves are centered around target interval, and the width of the distributions is proportional, as is characteristic of the scalar property of interval timing. This is the signature of the interval timing in adults (Gibbon, 1977; Rakitin et al., 1998).[Fig-anchor fig3]

## Discussion

This study provides the first direct measurement of infants’ interval timing abilities. Using an eye tracker, we examined the reaction of infants aged 4–14 months to the omission of a recurring target, on either a 3- or 5-s cycle. We found that infants at all ages showed a broadly similar pattern of responses. By analyzing both fixation data and pupil diameter changes, we attempted to dissociate voluntary from involuntary timing responses in infancy, based on the assumption that pupil dilation is involuntary and anticipatory eye gaze is under voluntary control. When examining the peak-to-peak variations on individual trials, we found evidence of interval timing ability in both measures. Specifically, we found that responses were approximately normally distributed about the target interval and the widths of the distributions were proportional to the length of the interval. These are the features of the scalar property in interval timing (Gibbon, 1977). To our knowledge, this has never been directly demonstrated in infants.

The scalar property is the key characteristic of interval timing. Timing is noisy, and errors grow linearly with the length of the interval. Previous studies have shown some interval timing abilities in infants, but their conclusions have been limited by methodological constraints. Habituation studies (e.g., Brannon et al., 2007; vanMarle & Wynn, 2006) only measure a binary response to a change in stimulus timing, whereas ERP measures (Brannon et al., 2008, 2007) only test for a miss-match negativity reaction to oddballs with very short intervals (<1,500 ms). Heart rate variability studies (e.g., Boswell et al., 1994; Colombo & Richman, 2002) sometimes show an anticipatory change to an omitted stimulus, but this response adapts rapidly. Our paradigm overcomes all of these limitations. Infants’ responses are measured during an omission period when nothing happens. Infants may generate timing predictions in the form of timed saccades to the target area. Or they may show involuntary responses to the task, as indexed by changing pupil diameter.

Developing a sense of timing is likely to involve the coordination of these explicit (gaze) control mechanisms with some means of internal time keeping. There is no direct means of assessing internal time keeping, but changes in pupil diameter might provide the best available signal. We found that pupil diameter changes were stronger predictors of the timing of the event than fixation; peak-to-peak changes in pupil diameter showed a much closer fit to Weber’s law (see Figure 3). But why might pupil diameter index timing ability? There is a well-established literature on using measures of pupil diameter to track preconscious or automatic processing in adults (see Laeng et al., 2012). Recent advances in analysis techniques suggest that changes in pupil diameter can show attentional effects (Wierda, van Rijn, Taatgen, & Martens, 2012), and attention is a key component of interval timing mechanisms (Block, Hancock, & Zakay, 2010). Research has shown that pupil diameter changes can indicate violation of certain physical expectations in 8-month-olds (Jackson & Sirois, 2009) and social expectations in 6- and 12-month-olds (Gredebäck & Melinder, 2010). In both of these cases, pupil diameter changes happen in response to an event occurring on the screen rather than in anticipation coupled with an unchanging screen, as we find here.

Research into infants’ volitional interval timing may also help address theoretical issues in the time perception literature. Addyman, French, Mareschal, and Thomas (2011) recently proposed a developmental model of interval timing in which there is an important role for embodied experience during the acquisition and calibration of time. Research with young infants may help decide between this type of developmental, embodied model and more traditional cognitive models of timing (e.g., Gibbon, Church, & Meck, 1984; Matell & Meck, 2004; Staddon & Higa, 1999). A growing body of research links time and space in adults (Dehaene & Brannon, 2010; Núñez & Cooperrider, 2013; Walsh, 2003); embodiment may provide a framework to account for this (Kranjec & Chatterjee, 2010). Accurate measures of individual variability in the subjective time estimation enables an exploration of the relationship between motor activity in infants and their subjective experience of time.

In summary, this study introduces a new method for investigating the interval timing abilities requiring the intrinsic generation of responses by young infants. Our results show that both voluntary and involuntary components of interval timing are present from 4 months of age. In particular, the errors generated by infants when anticipating the end of an interval follow the same scalar law as those observed in adults.

## Supplementary Material

10.1037/a0037108.supp

## Footnotes

1 Although some participants took part in both conditions, time interval was treated conservatively as between-subjects measure for all participants. Two-tailed *t* tests on the fixation and pupil data from the 3-s conditions showed no difference in group means (*p*s > 0.95) for infants who had or had not previously taken part in the 5-s condition.
