# Supplementary material for: Mapping the Origins of Time: Scalar Errors in Infant Time Estimation
Source: Dev Psychol. 2014 Jun 30;50(8):2030–5. doi: 10.1037/a0037108 (PMC4113309; doi:10.1037/a0037108)
Supplement: Supplementary file 1 [file DEV-DEV2-Addyman20132575-R-F1.FINAL.docx]

**Supplemental Materials**

**“Mapping the Origins of Time: Scalar Errors in Infant Time Estimation”**

**by C. Addyman et al., 2014, *Developmental Psychology***

**http://dx.doi.org/10.1037/a0037108**

For each infant we fitted smoothed curves to both the fixation and pupil data using piecewise smooth B-spline functions following the method described in Jackson and Sirois (2009; see also Ramsay & Silverman, 2002). This converted the data into a parametric form that allows us to produce average curves of each infant’s individual gaze and pupil response. Figure S1 shows the combined data of all the infants averaged across the three blocks of the experiment. Also shown on these plots are the periods when the target was present (blue bars) and when the target was omitted (red bars). The omission period was twice as long as a single cycle to allow us to capture the variability in infants’ time estimates (e.g., those cases in which infants took a relatively long time to look back). Infants’ looking and pupil diameter was closely time-locked to the animations during the familiarization phase but much more variable during the omission phase.

Figure S2 shows how individual time estimates were measured. For fixation data, experimenters manually marked the first and second minima in the period measured from the last appearance of the target until the end of the block. Time is measured as the distance between these two minima. For the pupil diameter, the distance was measured from first to second maxima, since appearance of the targets coincided with maxima in the infants’ pupil dilation. All peak-to-peak values were double coded by a second experimenter naïve to the hypothesis, interrater reliability was good (Cohen’s κ = 0.87).

Finally, because screen luminance can affect pupil diameter we calculated a relative luminance measure for each frame of the display based on the following formula:

Y = 0.2126 × R + 0.7152 × G + 0.0722 × B (1)

In this equation the raw RGB values of the input are combined with relative weightings corrected for the brightness perceived by a human observer (Poynton, 2003; Jackson & Sirois, 2009). For clarity, we scaled all of these values relative to the luminance of the empty frame (3.6 cd/m^2^). The averages for all target types are plotted as the thin red line in Figures S1B and S1D. This shows that, on average, the screen is fractionally brighter (+0.25%) when the target is present and momentarily darker (-0.43%) when the distractor first appears. It is unlikely that these small changes in overall brightness are driving the changes in pupil diameter. In any case, an increase in brightness would lead to a decrease in pupil diameter and the opposite pattern appears to hold here.


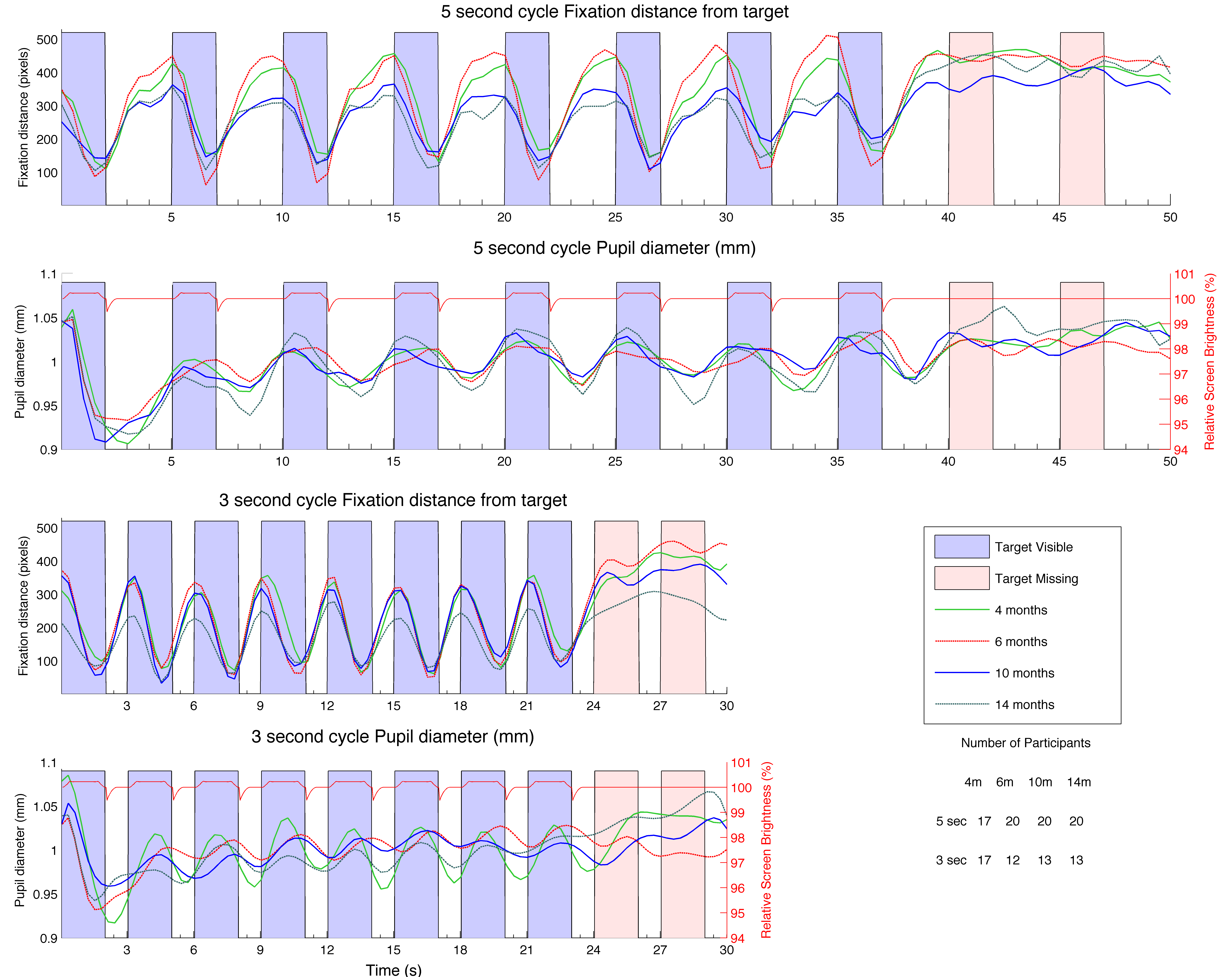


A

B

C

D

*Figure S1*. B-spline curves fitted to block data, averaged by age group (4, 6, 10, and 14 months) and time condition (3- or 5-second cycle). The shaded blue bars show when the target was on the screen, while the shaded red bars indicate the “missed beat” trial where the target did not appear. Panels A and C show the mean distance of infant fixation from the center of the target area, while B and D show the mean pupil diameter. Also shown in B and D is the mean change in relative perceived screen luminance plotted in red.


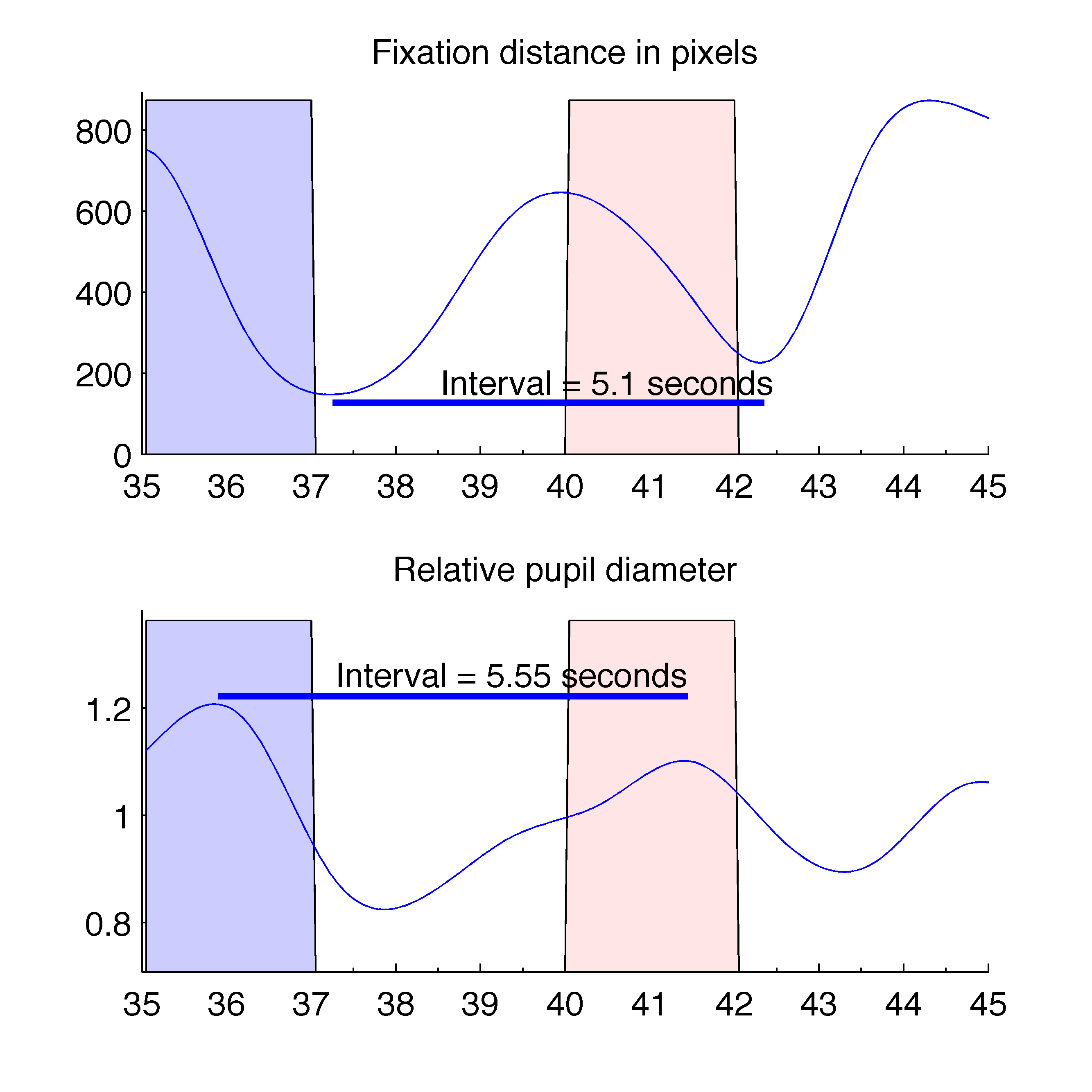


A

B

\

*Figure S2*. The blue lines illustrate how the peak-to-peak intervals present in the time series data were calculated, using minima for fixation distance (A) and maxima for pupil dilation (B). The shaded blue bars show when the target was on the screen, while the shaded red bars indicate when the target did not appear.
